# Combining genotypic and phenotypic variation in a geospatial framework to identify sources of mussels in northern New Zealand

**DOI:** 10.1038/s41598-021-87326-4

**Published:** 2021-04-14

**Authors:** Jonathan P. A. Gardner, Catarina N. S. Silva, Craig R. Norrie, Brendon J. Dunphy

**Affiliations:** 1 School of Biological Sciences, Victoria University of Wellington, Wellington, 6140 New Zealand; 2 School of Biological Sciences, University of Auckland, Auckland, 1142 New Zealand; 3 Present Address: Centre for Sustainable Tropical Fisheries and Aquaculture, College of Science and Engineering, James Cook University, 1 James Cook Dr, Townsville, QLD 4811 Australia; 4 Present Address: Hatfield Marine Science Center, Cooperative Institute for Marine Resources Studies, Oregon State University, Newport, OR 97365 USA

**Keywords:** Mass spectrometry, Biogeochemistry, Biooceanography, Ecology, Ecology, Environmental sciences, Ocean sciences, Marine biology, Genetics, Population genetics

## Abstract

The New Zealand green-lipped mussel aquaculture industry is largely dependent on the supply of young mussels that wash up on Ninety Mile Beach (so-called Kaitaia spat), which are collected and trucked to aquaculture farms. The locations of source populations of Kaitaia spat are unknown and this lack of knowledge represents a major problem because spat supply may be irregular. We combined genotypic (microsatellite) and phenotypic (shell geochemistry) data in a geospatial framework to determine if this new approach can help identify source populations of mussels collected from two spat-collecting and four non-spat-collecting sites further south. Genetic analyses resolved differentiated clusters (mostly three clusters), but no obvious source populations. Shell geochemistry analyses resolved six differentiated clusters, as did the combined genotypic and phenotypic data. Analyses revealed high levels of spatial and temporal variability in the geochemistry signal. Whilst we have not been able to identify the source site(s) of Kaitaia spat our analyses indicate that geospatial testing using combined genotypic and phenotypic data is a powerful approach. Next steps should employ analyses of single nucleotide polymorphism markers with shell geochemistry and in conjunction with high resolution physical oceanographic modelling to resolve the longstanding question of the origin of Kaitaia spat.

## Introduction

One of the biggest challenges marine biology has sought to address over the last 40 to 50 years has been to track the movement of larvae through the water column. Identifying source and sink populations and quantifying distance of dispersal as well as the rates and routes of migration amongst sites have been longstanding problems^1–3^. However, once acquired, such knowledge has helped to inform management in disciplines including fisheries, aquaculture, conservation and biodiversity protection, and more recently in understanding/forecasting the impacts of climate change^4–8^.

The sustainable management of many fisheries and also some aquaculture operations may depend on knowledge of the source of the individuals entering the fishery (i.e., being available for capture) or contributing to aquaculture (i.e., spat catching that contributes to ongrowing at farm sites)^8–10^. Identification of source populations, in particular in a coastal setting, may be critical to the longevity of the fishery or the aquaculture operation. However, because most marine larvae are so small and so many larvae are produced it is generally impractical to mark larvae at their origin and then to follow them as they move through the water column. This problem is compounded by the often very large spatial scale of realised and potential dispersal, its significant temporal variability, and its apparent ’chaotic ‘ patchy nature^11–13^. As a consequence, several different forms of ’natural tags ‘ have been employed to track individuals, most usually not the larval stage itself, but post-metamorphic stages such as juveniles or adults. Such tags have included genetic markers^14–16^, fish otoliths^17–19^, molluscan shell geochemistry^19–22^, and others such as lipid content^23^, stable isotopes^24–26^, and radioisotopes^27^. In most studies the different tagging methods have usually been used in isolation. However, improving power of resolution by combining two or more of these tagging methods is now a realistic option. A multi-marker approach may provide new insights into the spatial sources and temporal availability of larvae or spat, and the routes that they travel to reach settlement sites.

Farming of New Zealand’s endemic green-lipped mussel, *Perna canaliculus* Gmelin, 1791 is now the country’s most valuable aquaculture export. By 2017, total production was estimated to be ~ 100,000 tonnes pa, with a value of ~ NZ $350 M pa^28^. The industry has undergone rapid growth over the last 30 years, placing increasing demands on supplies of early stage juveniles, called spat^29,30^. The industry is largely reliant on wild caught spat, with ~ 80% of spat coming from Te Oneroa-a-Tōhē—Ninety Mile Beach (henceforth Ninety Mile Beach), in the far north^30^. In the New Zealand Quota Management System mussel spat may be collected by quota holders from area GLM9 which covers Ninety Mile Beach. Spat-bearing material is harvested after juvenile mussels are washed ashore attached to algae and hydroids^30^. A reliance on wild caught spat is a constraint to further industry growth, in particular given that the source populations that supply the spat are yet to be identified^21,29–31^. However, while hatchery production methods for this species are well established^32,33^, there remains a strong interest in the use of wild spat due to their ease of availability and low cost. To date, spat supply at Ninety Mile Beach has been surprisingly reliable and this availability has not hindered the expansion of the industry. However, in early 2019, spat supply to Ninety Mile Beach was low to non-existent for months at a time (Andrew Jeffs, University of Auckland, pers. comm.). More recently (early to mid 2020) spat supply has resumed, but there is still uncertainty around supply dynamics and a lack of knowledge of the locations of source populations.

The green-lipped mussel has a pelagic larval duration (PLD) of approx 28–35 days depending on environmental conditions and season, produces a planktotrophic (feeding) larval stage that stays near the water column surface, and which is generally a passive particle (i.e., there is very limited to no active swimming capability)^30^. The coastal and offshore physical oceanography of the northwest of New Zealand is poorly understood^30^. The most recent study of the region indicates that, in general, flows are weak and highly variable^31^. Offshore, beyond the 1000-m isobath, the mean flow is to the southeast, whereas inshore of the 1000-m isobath and close to the coast the mean flow is northwestward^31^. Examination of a long-term data set revealed that the supply of Kaitaia spat is positively associated with strong offshore winds, with low swell height in the onshore direction, and with the number of storm events per year^32^. Overall, these observations^30–32^ suggest that the natural supply of mussel spat to Ninety Mile Beach is a complex interaction of numerous different biological, physical oceanographic and meteorological factors.

Assessments of PLD and coastal physical oceanography suggest that spat arriving at Ninety Mile Beach may be derived from one or more source populations to the south, in the region from Hokianga Harbour to Taranaki^30^. Previous work^33^ using traditional genetic clustering approaches failed to resolve any genetic structure (differentiation) amongst mussels collected from this northern region, whilst analyses of shell geochemistry revealed large differences amongst mussels from different sites^21,22,33^. The identification of source populations has remained problematical. Striking a balance between what at present appear to be uninformative microsatellite data and perhaps overly variable shell geochemistry data seems to be necessary if our knowledge of source populations is to be advanced, but is proving to be a difficult next step. However, using a new analytical method that combines genotypic with phenotypic information, and placing these in a geospatial context^34^, may provide new insights into the origin of Kaitaia spat at Ninety Mile Beach.

In the present paper we describe geospatial analyses of separate and combined genotypic and phenotypic data sets of green-lipped mussels collected from six sites in northern New Zealand. Two of our sites (Scott Point at the northern end and Ahipara at the southern end of Ninety Mile Beach) are representative of Kaitaia spat, the mussels used by industry. We use these samples as our ‘target’ mussels. The four other sites (listed north to south), Tanutanu Beach, Mitimiti, Whatipu and Oakura Beach, are representative of sites that may be sources of Kaitaia spat. We use these samples as our putative ‘source’ mussels. These four sites span ~ 640 km of coastline to the south of Ninety Mile Beach. Our testing of ‘target’ and ‘source’ mussels is subject to some possible error or bias because pronounced temporal variation in spat supply (i.e., different sites contributing spat to Ninety Mile Beach at different rates and/or at different times) may result in loss of signal definition for this work. To try to mitigate this we used mussels of different sizes (ages) to reduce the likely affect of one large atypical pulse of spat arriving at Ninety Mile Beach. That is, we have taken a time-averaged sample of mussels (mussels of different ages). Additionally, mussels were collected from Ahipara in January, February and March of 2015 for shell geochemistry analyses to better understand the extent of temporal variation in Kaitaia spat supply. We quantified genotypic and phenotypic variation amongst mussels from these six sites, and then analysed these combined data sets in a geospatial framework that allows for correlated alleles, for spatial testing, and for inclusion of null alleles^34^, to better understand how powerful this approach is as a method for identifying putative source populations.

## Results

In total, genotypic (microsatellite variation at 10 polymorphic loci) data were collected for 288 mussels from six sites and phenotypic (shell geochemistry variation of 12 elements) data from 395 mussels from six sites, with one site having three temporal samples. Mussels collected from Scott Point (SCO) and Ahipara (AHI) were expected to be most like the spat collected for the aquaculture industry from the region of Ninety Mile Beach, whereas mussels from the four southern sites (TAN, MIT, WHA, OAK) are representative of putative natal populations from which the Ninety Mile Beach mussels may be derived.

### Microsatellite variation

Micro-Checker identified putative null alleles at five loci—*Pcan1-27*, *Pcan1-29*, *Pcan6-17*, *Pcan10-44* and *Pcan22-11*. No long allele dropout was detected. After false discovery rate (FDR) correction, *Pcan1-27* was identified as being significantly out of HWE at all populations and *Pcan10-44* at more than half of the samples. No evidence of significant linkage disequilibrium was detected between locus pairs. Site-specific values of mean number of alleles, observed and expected heterozygosities, *F*_IS_ and also *A*_P_ were all very similar across sample locations (Table 1; Table S2).

**Table 1:** Descriptive measures of genetic variation in *Perna canaliculus* for 10 microsatellite loci and six site samples.

Site	*N*	*N*a	*H*_O_	*H*_E_	*F*_IS_	*A*_P_
Scott Point	50	11.00 ± 2.37	0.64 ± 0.05	0.73 ± 0.05	0.13 ± 0.04	5
Ahipara	50	10.80 ± 2.24	0.64 ± 0.05	0.72 ± 0.05	0.10 ± 0.07	5
Tanutanu	50	10.40 ± 1.89	0.63 ± 0.05	0.71 ± 0.05	0.10 ± 0.07	3
Mitimiti	47	10.90 ± 2.11	0.67 ± 0.06	0.75 ± 0.04	0.09 ± 0.08	5
Whatipu	41	10.40 ± 2.02	0.64 ± 0.06	0.72 ± 0.05	0.10 ± 0.05	3
Oakura	50	10.30 ± 1.80	0.62 ± 0.06	0.70 ± 0.06	0.11 ± 0.06	4

Number of mussels (*N*), mean number of alleles per site (*N*a), observed heterozygosity (*H*_O_), expected heterozygosity (*H*_E_), the fixation index (*F*_IS_) and the number of private alleles across all loci (*A*_*P*_). Sites are arranged north to south.

*N* = number of mussels analysed; *Na* = number of alleles per locus (mean ± SD); *H*_*O*_ = observed heterozygosity; *H*_*E*_ = expected heterozygosity; *F*_*IS*_ = fixation index; *A*_*P*_ = total number of private alleles.

Allelic (genic) differentiation was observed in 9 of 15 pairwise sample comparisons after correction for multiple testing (Table 2). Overall, across all 10 loci, a significant difference in allelic frequencies was observed amongst mussels from the six sites (χ^2^ = 57.5, *DF* = 20, *P* < 0.001). Genotypic differentiation was observed for only 1 of 15 pairwise comparisons, after correction for multiple testing (Table 3). Overall, across all 10 loci, a non-significant difference in genotypic frequencies was observed (χ^2^ = 29.3, *DF* = 20, *P* = 0.082).

**Table 2:** Significance (*P*-values) levels for differentiation between each site pair across all loci (Fisher's method).

	Scott Point	Ahipara	Tanutanu	Mitimiti	Whatipu	Oakura
Scott Point	–	0.059	0.451	0.639	0.551	0.953
Ahipara	**0.003**	–	0.096	**< 0.001**	0.127	0.088
Tanutanu	0.166	**0.008**	–	0.178	0.805	0.759
Mitimiti	0.316	**< 0.001**	**0.020**	–	0.127	0.055
Whatipu	0.310	**0.014**	0.546	**0.007**	–	0.337
Oakura	0.639	**0.015**	0.344	**0.004**	**0.087**	–

*P*-values for allelic differentiation across all 10 loci below the diagonal and for genotypic differentiation across all 10 loci above the diagonal. Values in bold are statistically significant after correction for multiple testing. Sites are arranged north to south.

**Table 3:** Pairwise *F*_ST_ values for the six samples of *Perna canaliculus* assessed over all 10 loci.

	Scott Point	Ahipara	Tanutanu	Mitimiti	Whatipu	Oakura
Scott Point						
Ahipara	**0.001**					
Tanutanu	0.000	**0.000**				
Mitimiti	0.001	**0.009**	**0.007**			
Whatipu	0.000	**0.000**	0.000	**0.004**		
Oakura	0.000	**0.002**	0.000	**0.011**	0.005	

Negative *F*_ST_ values have been set to zero.

Significant values of *F*_ST_ after FDR testing are in bold.

Pairwise estimates of *F*_ST_ were low (0.000–0.011, mean ± SD = 0.003 ± 0.004) (Table 3). Eight of 15 *F*_ST_ values were statistically significant after correction for multiple testing.

### Population genetic structure

**Figure 1:**
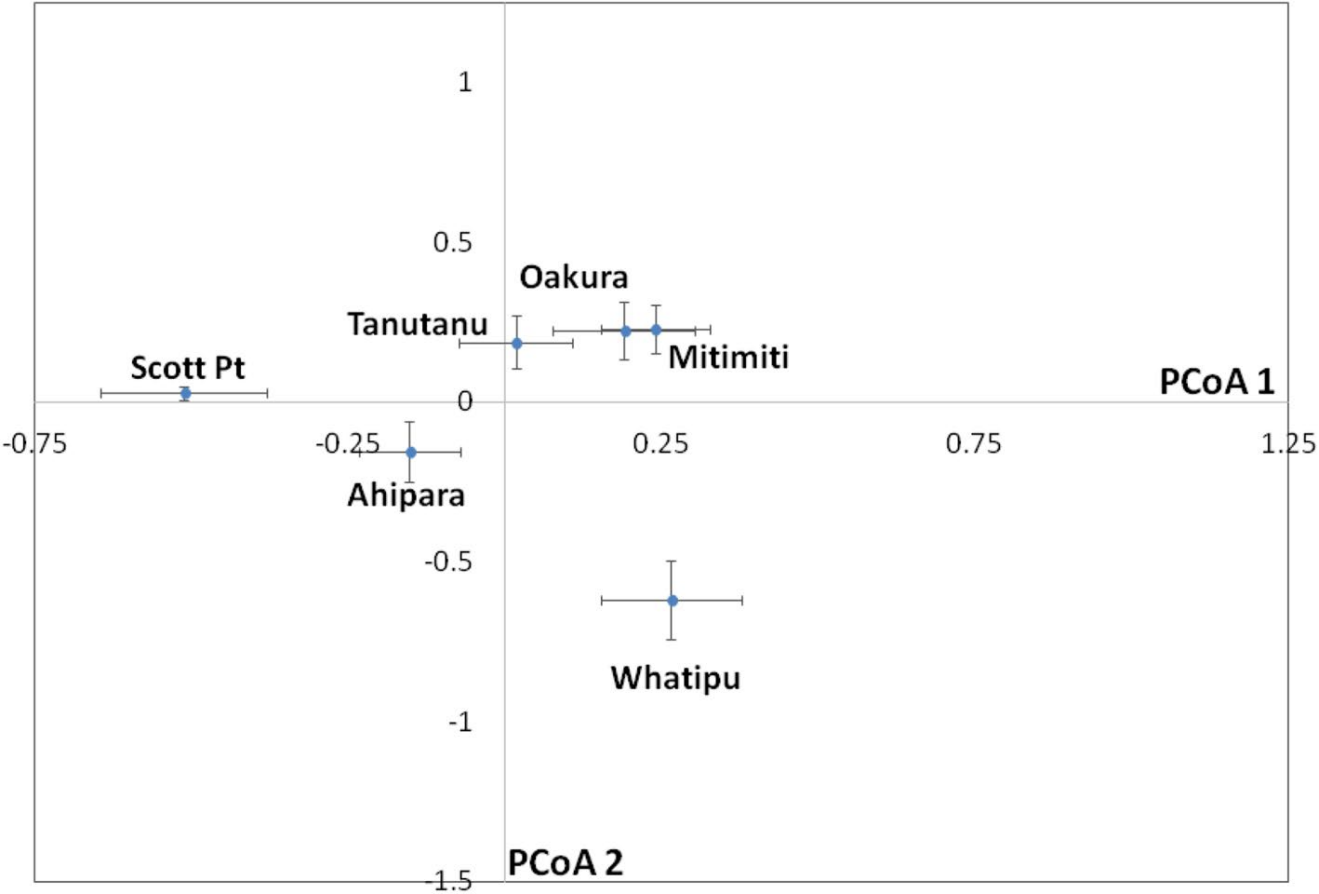
Principal Coordinates analysis (PCoA) plot (site-specific means ± SE) for six samples of *Perna canaliculus* based on variation at 10 microsatellite loci.

The PCoA plot of multilocus microsatellite variation (site-specific means ± SE) revealed clear separation amongst most samples (Fig. 1). There was no pronounced separation of samples based on geography, although the two northern-most sites (Scott Point, Ahipara) were both located to the left of the plot (negative PCoA 1 values). Three sites (Tanutanu, Mitimiti, Oakura—listed north to south) were located close to one another with some degree of overlap on both the x and y axes. The Whatipu sample (mid-point of our sampling range) was differentiated from all other samples.

### Assignment testing

Mean ± SD assignment probabilities for mussels from the six sites were 49.8% ± 18.5 (Table S3a). The two most northerly sites of Scott Point and Ahipara (expected to be recipient sites of mussel spat washed up at Ninety Mile Beach) had two of the three lowest assignment values. Mussels of the four northern-most sites all had total assignment values of ~ 55% (mean ± SD = 56.65 ± 7.01), whereas mussels from the two most southerly sites, which are 300 km and 600 km south of the main spat collection region, had overall assignment values of 79% (Whatipu) and 53% (Oakura).

Only 12 first generation (*F*_0_) migrants were identified. Scott Point contributed and received the greatest number of *F*_0_ migrants (4 and 3, respectively), whereas Tanutanu contributed no *F*_0_ migrants and, along with Ahipara and Oakura, received only one migrant each (Table S3b).

### Geochemical marker variation

Fifty shells from each site were processed and analysed. However, due to suspected metal contamination only 49 shells from Scott Point and Ahipara (Jan) and 47 for Ahipara (Feb) were used in subsequent statistical analyses.

Running the Q-DFA with a step-wise procedure revealed that six elemental ratios (B:Ca, Co:Ca, Li:Ca, Mg:Ca, Mn:Ca, Ni:Ca) explained the majority of the classification success (96.7%) of the Q-DFA (Table S4). These results were supported by the overall per cent classified figures of the Q-DFA model whereby 92–100% of shells were classified to their site of collection when using the above six ratios. The addition of further elemental ratios i.e., Ba:Ca, Cu:Ca, Sr:Ca, Ti:Ca and Zn:Ca, provided marginal to no improvement in classification success (Table S5).

Whilst the ability of the Q-DFA to correctly assign mussels to their site of collection varied from sample to sample, classification success was high (Table 4). Mussels collected from Whatipu and from Ahipara in February and March were correctly assigned to their site of collection with 100% classification success, whereas mussels from the remaining sites (Ahipara in January, Scott Point, Tanutanu, Mitimiti and Oakura) were correctly classified to their site of collection with between 92 and 98% success. Typically, misclassified shells were assigned to sites immediately geographically adjacent to the actual collection site (Table 4), the exception being Oakura with shells assigned to Scott Point and Ahipara. The Receiver Operating Characteristic (ROC) curves showed a high degree of sensitivity for this model, with values in the range 0.995–1.000 (Table 4).

**Table 4:** Classification success of a quadratic discriminant function analysis (Q-DFA) model run on elemental ratios in the shells of juvenile green-lipped mussels (*P. canaliculus*) collected in January 2015 from six sites along the west coast of the North Island of New Zealand.

Predicted collection site											
Collection site	Scott Pt	Ahipara Jan 15	Ahipara Feb 15	Ahipara Mar 15	Tanutanu	Mitimiti	Whatipu	Oakura	n analysed	% Classified correctly	ROC area
Scott Pt	**47**	1	0	0	0	0	0	1	49	96	0.9983
Ahipara Jan 15	2	**45**	1	0	0	0	0	1	49	92	0.995
Ahipara Feb 15	0	0	**47**	0	0	0	0	0	47	100	0.9979
Ahipara Mar 15	0	0	0	**50**	0	0	0	0	50	100	1.0000
Tanutanu	0	1	0	0	**49**	0	0	0	50	98	1.0000
Mitimiti	0	1	0	1	0	**48**	0	0	50	96	0.9999
Whatipu	0	0	0	0	0	0	**50**	0	50	100	1.0000
Oakura	1	1	2	0	0	0	0	**46**	50	92	0.9972
Total	50	50	50	51	49	48	50	48	395	96.7	0.9964

Additional mussels were collected from Ahipara in February and March 2015. Sites listed from north to south.

Numbers in bold, on the diagonal, indicate the number of mussels from a collection site that were correctly classified as being from that site (the predicted collection site).

**Figure 2:**
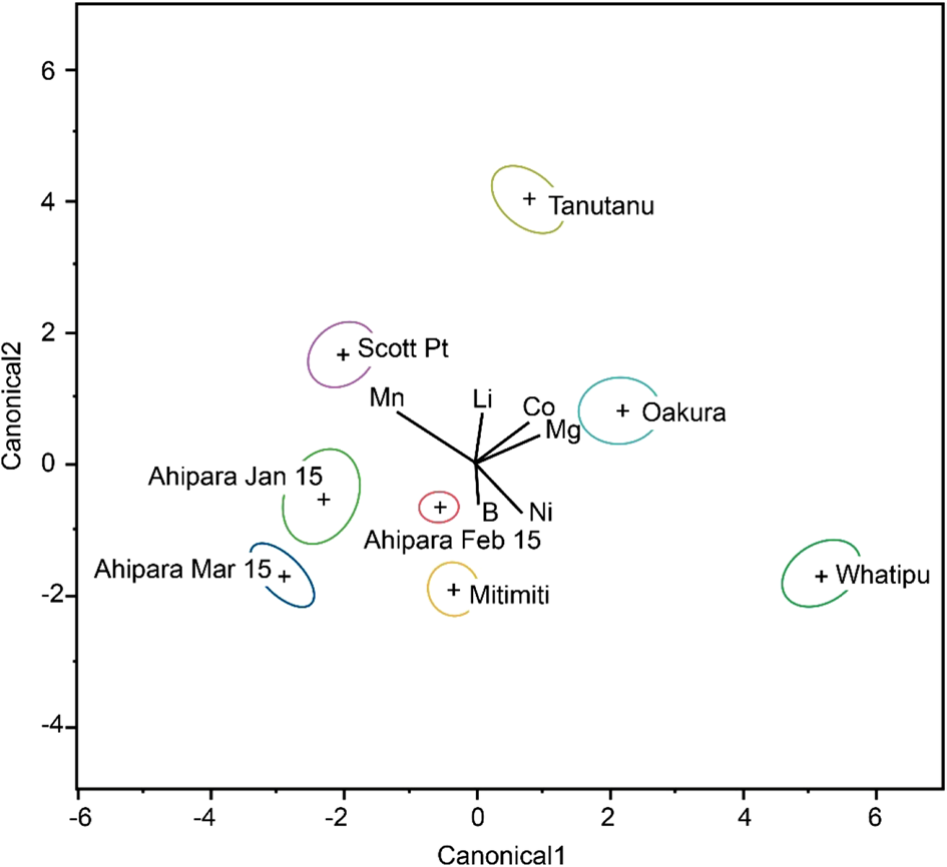
Average (cross symbol) and 95% confidence intervals (coloured ellipses) of canonical scores from quadratic discriminant function analysis of ratios of *TE*:Ca to B, Co, Li, Mg, Mn and Ni in shells of juvenile *P. canaliculus* collected from six sites within the North Island of New Zealand in January 2015. To gain an understanding of temporal variation samples were also collected in February and March at Ahipara. Direction of spread of site-specific samples is explained by elemental directions shown in the centre of the plot.

The plot of the Q-DFA canonical scores revealed no overlap of the 95% confidence interval ellipses for the ratios of B, Co, Li, Mg, Mn and Ni to Ca in shells collected in January 2015 from the six sites, as well as the additional temporal samples collected from Ahipara in February and March, 2015 (Fig. 2). These results highlight how pronounced elemental variation contributes to differentiation of the spatial and temporal samples.

**Figure 3:**
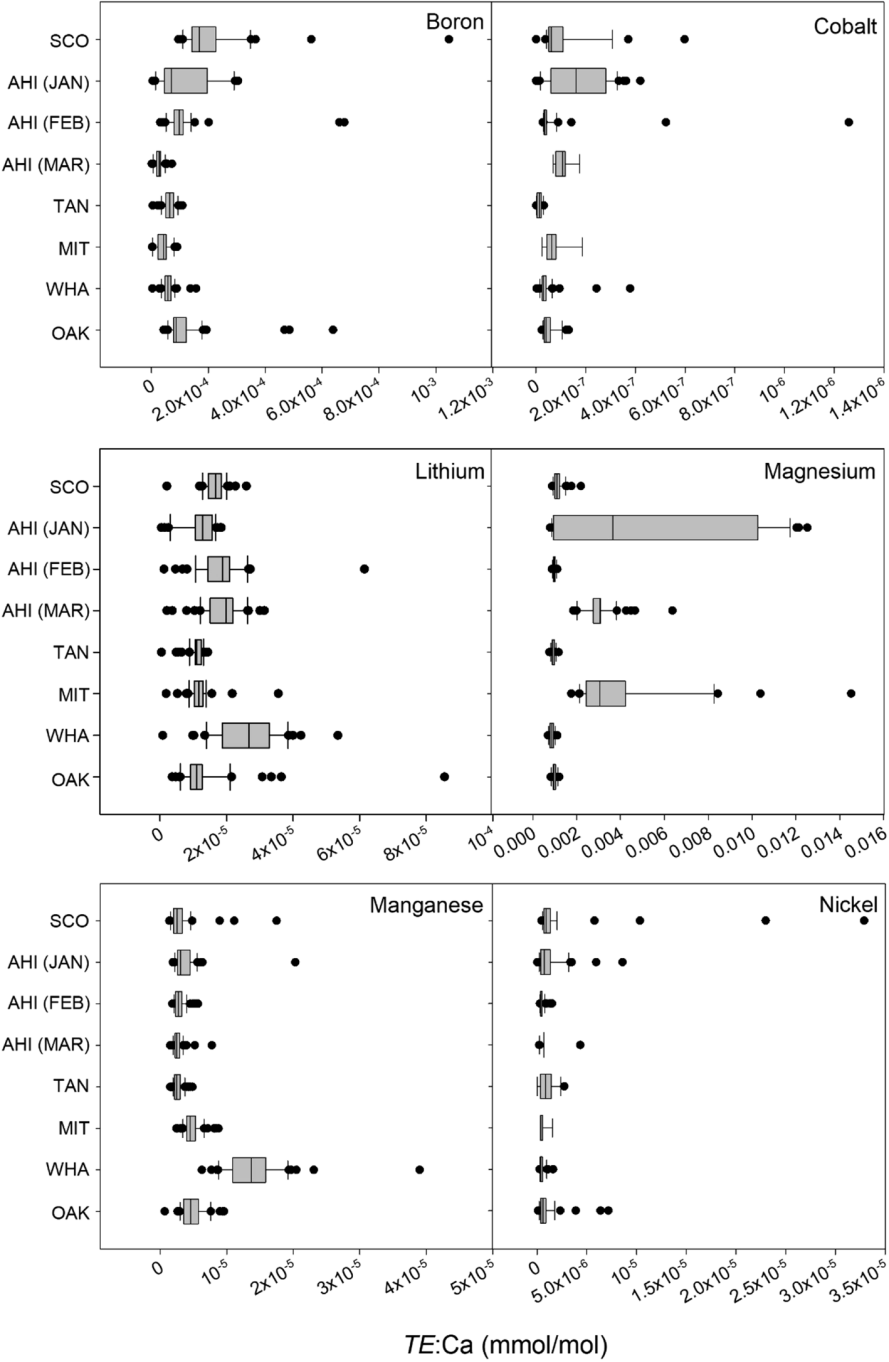
Boxplots of elemental ratios (*TE*:Ca on x-axis) quantified via ICP-MS in shells of juvenile green-lipped mussels (*P. canaliculus*) collected from Scott Point (SCO), Ahipara (AHI, Jan, Feb, Mar), Tanutanu Beach (TAN), Mitimiti (MIT), Whatipu (WHA) and Oakura (OAK) in 2015. Sites are displayed from north to south on the y-axis. Solid lines within the box indicate medians, boxes and whiskers indicate the 10th, 25th, 75th and 90th percentiles, respectively.

Ratios of elements to Ca within the shells varied significantly amongst the sites (Fig. 3; Table S6) for multiple comparisons following Kruskal–Wallis tests. The ratio for Boron was significantly higher in shells from Scott Point (*H*_(1,7)_ = 253.9, *P* < 0.001) than at other sites. Lithium (*H*_(1,7)_ = 190.1, *P* < 0.001) and manganese (*H*_(1,7)_ = 230.2, *P* < 0.001) ratios were significantly higher in shells from Whatipu than all other sites. Cobalt ratios were significantly higher in Ahipara mussel shells in January 2015 (*H*_(1,7)_ = 248.4, *P* < 0.001) but not in February or March. Finally, the ratio for nickel was significantly higher in shells from Tanutanu (*H*_(1,7)_ = 162.7, *P* < 0.001) than in shells from all other sites, and although magnesium was highly variable in shells collected from each site (particularly Ahipara in January), it was greatest in Mitimiti shells (*H*_(1,7)_ = 259.2, *P* < 0.001). These results highlight the spatial (all six sites) and temporal (three samples from Ahipara) variation that exists in shell geochemistry of green-lipped mussels.

### Geneland results

#### Genotypic variation in a geospatial context

In 20 independent runs Geneland resolved *K* = 2 (4 times), *K* = 3 (12 times) and *K* = 4 (4 times), where *K* is the number of clusters, based on genotypic variation of the mussels from the six sites. The top four models were *K* = 3 (twice) and *K* = 4 (twice). These results indicate that Geneland cannot resolve the *K* = 3 or *K* = 4 question, that the most likely interpretation is *K* = 3, but also that the mussels from the six sites do not constitute one homogenous group. Whatipu was placed into its own cluster in 16 runs, Scott Point in 14 runs, and Mitimiti in six runs. Ahipara, Oakura and Tanutanu clustered together in 18 of 20 runs. Pairwise *F*_ST_ values were low and ranged from < 0.001 to 0.036 (mean ± SD = 0.018 ± 0.013, n = 64) (Table S6).

#### Spatial phenotypic variation in a geospatial context

In 10 independent runs Geneland resolved *K* = 6 in every instance. Based on shell geochemical variation the mussels from all six sites were very different and could easily be differentiated.

#### Temporal phenotypic variation in a geospatial context

In 10 independent runs Geneland resolved *K* = 3 in every instance for the monthly samples (January, February, March 2015) of mussels from Ahipara. Likewise, in 10 independent runs Geneland resolved *K* = 8 in every instance when all eight samples (i.e., six spatial samples collected in January 2015 plus the February and March 2015 samples from Ahipara) were analysed together. These results indicate that geochemical differences amongst shells collected from Ahipara at monthly intervals are sufficient to permit identification of month-specific samples: there is pronounced temporal variability in shell geochemical signatures.

#### Combined genotypic and phenotypic variation in a geospatial context

In 10 independent runs Geneland resolved *K* = 6 in every instance. These results indicate that the mussels from all six sites are very different.

## Discussion

Analysis of genotypic and phenotypic variation in a geospatial framework^34^ may help with the identification of previously undetected clusters or the novel identification of source populations. The ability to utilise both phenotypic and genotypic information in assignment testing is important because phenotypic variation may be high even when genotypic variation is low within the same individuals. This phenomenon is often reported^35,36^ and may occur because the genetic markers being used do not adequately reflect phenotypic variation or because genotypic variation and phenotypic variation are influenced by different processes^37^. Thus, given the differing levels of information they can provide, a combined genetic and geochemical approach may be particularly informative in identifying green-lipped mussel source populations. This approach has been used to delineate stocks of Mediterranean hake (*Merluccius merluccius*) and it was reported that microsatellite methods provided broad scale geospatial information of gene flow on evolutionary timescales, whereas geochemical methods could provide fine scale geospatial information on ecological relevant timescales^38^.

In terms of larval connectivity, new mussel recruits to any site are expected to originate from source sites to the south: given the weak mean northwestward flow of water along the coast^31^ and the 28–35 day PLD^32^ the natal sites could be 10s to 100s of km away. Balanced against this are the high levels of self-recruitment (in the range 40–70%) reported for green-lipped mussels^39^, and in addition factors such as offshore winds, storminess and low swell height in the onshore direction will all contribute to variation in natural spat deposition on Ninety Mile Beach^32^. Previous work in the North Island of New Zealand (including in the far north region) has been unable to resolve any population genetic structure (spatial differentiation) despite using a range of different genetic marker types^33,39–41^. These studies did not employ geospatial analyses, and the apparent absence of spatial genetic differentiation suggested that it would be very hard, if not impossible, to identify source populations for the Kaitaia spat, given that all mussels in the region belonged to one homogeneous ‘northern’ group. The new analyses reported here resolve genetic differentiation between/amongst mussels from six sites in this northern region. Whilst different results were resolved by Geneland for the genotypic data alone, the most frequently reported was *K* = 3 clusters, with the sites of Ahipara, Mitimiti, Oakura and Tanutanu forming one group, and the sites of Scott Point and Whatipu each forming separate groups. There is no obvious association between a site’s geographic location and its group membership. In terms of determining the origin of spat collected from the Ninety Mile Beach sites (Scott Point in the north, Ahipara in the south) these results indicate that we have not collected source mussels for the spat arriving at Scott Point, but that spat arriving at Ahipara may be derived from two neighbouring sites of Tanutanu and Mitimiti, which are 15 km and 48 km south, respectively, and also from the most distant site of Oakura, which is ~ 640 km south. Mussels from the northernmost Kaitaia spat site of Scott Point were consistently identified as being genotypically distinct, as were mussels from the geographically intermediate site of Whatipu, which suggests that both receive limited larval supply (gene flow) from the other four sites. Pairwise *F*_ST_ estimates amongst the *K* = 3 clusters confirm this interpretation. Based on the microsatellite markers alone, these results indicate that Kaitaia spat are most probably not derived from a single source population/site, but are a mix of mussels derived from multiple different sites. The estimate of the total number of first generation migrants amongst all sites was 12 of 288 individuals analysed. Estimates of the number of first generation migrants between pairs of sites were all in the range 1 to 3. The two Kaitaia spat sites of Scott Point and Ahipara had the highest (n = 3) and the lowest (n = 1) number of first generation migrants, respectively. When grouped according to the Geneland results, the number of first generation migrants was 4 for Ahipara, Tanutanu, Mitimiti and Oakura, 3 for Scott Point, and 3 for Whatipu. There is no obvious geographic explanation for the patterns of gene flow (source populations versus destination sites) revealed by these analyses, but for the first time we have evidence that microsatellite variation can be used to accurately differentiate amongst samples collected 10s to 100s km apart within what was previously recognised as one large genetically homogenous ‘northern’ group^33,39–41^.

Analysis of the shell geochemistry of mussels collected from the six sites revealed very high levels of elemental variation. In total, 17% (739 of 4396) of analysed geochemical values were imputed which could potentially introduce bias in the analysis. However, there was no correlation between importance of an element within the Q-DFA and the level of imputation (unpub. data). Assignment test success results were ~ 95% (range 90–100%), and are much higher than those reported for the same mussels when based on genotypic variation (mean ~ 50%, range 30–79%). High levels of assignment success based on shell geochemistry suggest that mussels from each site experience environmental conditions that result in different shell chemical fingerprints. Geneland recognised *K* = 6 clusters (i.e., mussels from each site were different from all other sites) in every run for the shell geochemistry data, confirming the distinct nature of the six samples. However, the PCoA plot revealed that shells from Scott Point and Ahipara (the sites at Ninety Mile Beach) exhibited the greatest variation in shell geochemistry. Thus, mussels from sites that are only a few kilometres apart can be differentiated with a very high degree of confidence. Clearly, this finding needs to be tested against samples from other sites to determine the extent of site specificity versus some sort of regional specificity. In a management sense, when trying to trace the source of Kaitaia spat, such high levels of shell geochemical variability are really only informative when mussels collected from Ninety Mile Beach cannot be differentiated from mussels collected from another site (the putative source) because absence of differentiation implies similarity of mussels from source and recipient sites. To resolve mussel sources would require high resolution subsampling of adult shell at the umbo using laser ablation ICP-MS. The composition of the umbo represents larval shell geochemistry which can be compared to the spatial variation in overall shell geochemistry shown in this study. Recently, this approach was used to determine level of larval spill over from mussel aquaculture to adjacent mussel beds^42^. Future studies using LA-ICP-MS analyses of shells along the northwest coast of New Zealand are planned to address this.

Geneland analysis of combined genotypic and phenotypic variation revealed *K* = 6 clusters, with each site-specific sample being different from all other samples. That is, the addition of the genotypic data set to the phenotypic data set did not increase the power of the analysis to detect differences amongst the clusters. At the moment, our analyses suggest that shell geochemical signal is more informative than the genetic signal in assigning mussels to site. Nonetheless, we view the combined application of genotypic and phenotypic data sets to be an important tool and a major step forward in terms of information (e.g., if phenotypic and genotypic traits are under different selection regimes) and management options^43^. This will be particularly true when the next generation of molecular markers is developed and employed (see subsequent section).Whilst we may not have been able to identify the source population(s) of Kaitaia spat our results indicate that combined genotypic and phenotypic data sets, when analysed in a geospatial framework, probably have the necessary discriminatory power to identify source populations. However, it is equally apparent that these analyses are, to a large extent, only as good as the source sites that are sampled. In the present study we focussed on six sites, two of which represent the Kaitaia spat collecting region and four of which are further south and could reasonably have been sources. Subsequent work will require more focussed spatial coverage of sample sites, and ideally this work will be a targeted approach to site selection based on, for example, physical oceanographic models^8^ that can be used to help identify source populations, rather than the haphazard site selection process involved here. Subsequent work may also be informed by our results. For example, mussels from Whatipu were identified consistently as being different from all other mussels. This may be because the site’s location on Manukau Harbour, a large body of seawater contained in a coastal embayment to the west of the city of Auckland, is expected to be characterised by a chemical composition that is very different from that of all other sites sampled. The results from Whatipu are helpful in that they suggest that further detailed examination of the geochemistry signals in the shells of mussels from Scott Point and Ahipara may permit the identification of environmental signatures that can be used to help identify source sites.

Six elemental ratios (Mg, B, Mn, Li, Ni, Co:Ca) provided the majority of classification success in discriminating amongst the six west coast mussel samples along the northern North Island of New Zealand. These six elemental ratios assigned mussels to their site of origin with 92–100% success: this information is valuable because it limits the number of elements needing to be assayed in future geochemical tag studies on this species along this coast. Reliable discrimination of mussels from sites encompassing similar distances to those in our study (10s-100s of km) have been recorded using geochemical tags for a number of marine taxa, including blue mussel (*M. edulis*) populations at ~ 50 km distance^44^, populations of *M. californianus* and *M. galloprovincialis* 20 km apart^20^, and New Zealand green-lipped mussel populations at a distance down to 12 km^21^. These studies highlight the power and utility of the shell geochemistry approach in identifying source populations, and also in addressing questions of spatial and temporal settlement variability at ecological relevant scales.

Populations occupying estuaries or those near streams or rivers are often easily discriminated using geochemical tags^20,45^ because localised terrigenous input of elements within freshwater is important in controlling the availability of at least some elements^46^. Two of our sites, Oakura and Whatipu, are located near sources of freshwater and were readily distinguished from other sites. Although there was a tendency for a small number of mussels to be assigned to Oakura despite being 640 km to the south, mussels from the remaining sites were also able to be discriminated from one another with equal if not greater success. One explanation for this could be that the west coast of the North Island of New Zealand is an area of active terrigenous sedimentation due to the active plate boundary running through New Zealand^47^. This results in considerable uplift with erosion through a varied mix of volcanic and sedimentary rock catchments^48,49^. In the far north, the geology comprises a complex mix of allochthonous igneous rock, Miocene sedimentary rock and mixed accumulated sediments from the Pleistocene and Holocene periods^50^. Accordingly, it may well be that the elemental composition of surrounding coastal waters is complex due to being fed by riparian inputs draining a complex geological landscape.

Understanding the spatio-temporal stability of geochemical tags is vital if such tags are to be informative. Some species, such as the cockle, *Cerastoderma edule*, demonstrate significant temporal variability in geochemical tags among years^51^, whereas other species show seasonal stability in geochemical tags e.g., the hardshell clam, *Mercenaria mercenaria*^52^. Analysing geochemical tags in mussels collected from Ahipara on a monthly timeframe (January, February, March, 2015) revealed that mussels could be assigned back to their site of origin (Ahipara) with a high degree of confidence (0.995–1.000), both in the context of the other five sites and also in terms of the month of collection (i.e., spatially and temporally). There was, however, a comparatively high degree of variability in elemental ratios (e.g. Mg:Ca) amongst the mussels of the three-monthly samples. Comparing shell lengths of mussels sampled during these three months reveals a significant increase in shell length between January to February (one-way ANOVA *F*_(2,149)_ = 5.931, *P* < 0.003), which presumably contributed to differential incorporation of elements into growing shells. The three samples from Ahipara illustrate the extent of short-term temporal variability in elemental composition (e.g., cobalt ratios were significantly higher in Ahipara shells collected in January than in February and March) at any one site. Such variation may be highly informative in identifying spat source populations, but only as long as this shell geochemistry signal can be linked back to the source population(s).

The shell geochemistry data in particular, and the population genetics data to a lesser extent, highlight spatial variability (and temporal variability for the geochemistry) that may, in the future, be informative in identifying Kaitaia spat source populations for the New Zealand green-lipped mussel industry. Temporal variation in geochemistry likely reflects changing environmental conditions on short temporal timeframes at the sampling sites. These results present a challenge to the future identification of the source populations, given that the geochemical and genetic signals of the spat at Ninety Mile Beach will be mixed, reflecting the differing spatial and temporal contributions from different source populations. Interpreting this mixed signal will be difficult, but not impossible, and is akin to the use of mixed source models employed to test the origin of stable isotopes in diet analysis^25,26^.

The ICP-MS method is an accurate approach to be able to discriminate populations separated by as little as 5 km along an open coast. In this study, the elemental ratios of B, Co, Li, Mg, Mn, and Ni:Ca appear to consistently discriminate mussel populations along the west coast of northern New Zealand. It is now vital that the spatio-temporal stability of these geochemical tags along this coastline be confirmed. Furthermore, if determining provenance of harvested mussels is of interest then shell geochemistry has a demonstrated role to play here as has been shown for other bivalve species e.g. *Mytilus edulis*^53^. The use of LA-ICP-MS methods combined with a thorough sampling regime (both temporally and spatially) will be necessary to trace green-lipped mussel spatfalls back to their site(s) of origin.

From a management perspective the identification of the Kaitaia spat source populations remains important, even as the industry moves towards commercial-scale hatchery production of spat. Hatchery production will help to secure year-round availability of spat and will permit selective breeding of spat with fitness characteristics of interest to the industry^54,55^. However, in the meantime, the supply of spat to Kaitaia that underpins the New Zealand green-lipped mussel aquaculture industry remains unpredictable, even if it is reasonably cheap to collect and distribute around the country. It seems likely that over the next several decades many mussel growers will continue with Kaitaia spat as their preferred source of juvenile mussels for stocking their farms. In addition, Aquaculture NZ has plans to expand coastal and open water aquaculture in New Zealand, and a large part of this strategy is focussed on growth of the green-lipped mussel sector^56^. Where the spat supply to meet this demand will come from is unclear, again suggesting that Kaitaia spat will remain an important resource for the green-lipped mussel aquaculture in New Zealand in the foreseeable future.

Future research to identify source populations of Kaitaia spat will include the use of more informative genetic markers (single nucleotide polymorphisms, SNPs) than the microsatellite markers employed here. SNPs are highly polymorphic co-dominant markers that can now be developed reasonably cheaply and in very large numbers (thousands to hundreds of thousands). They have proven to be useful in a range of different approaches^57–59^ and are expected to outperform microsatellite markers for information content. This is expected to translate into far greater power to discriminate amongst samples in a spatial and temporal context. In addition, the results of the present study highlight the need for a more focussed approach to site selection. Future work will therefore be guided by newly developed high definition physical oceanographic models that will provide much more guidance for site selection than was available to us at the time of this study. Finally, Norrie et al. (2019)^22^ analysed shell geochemistry in selectively bred green-lipped mussel and demonstrated a genetic component in the incorporation of elements within the shell of this species. If such population differences could be uncovered in wild mussels this could further strengthen future analyses seeking to determine site of larval origin.

## Materials and methods

### Collection and processing of juvenile mussels

**Figure 4:**
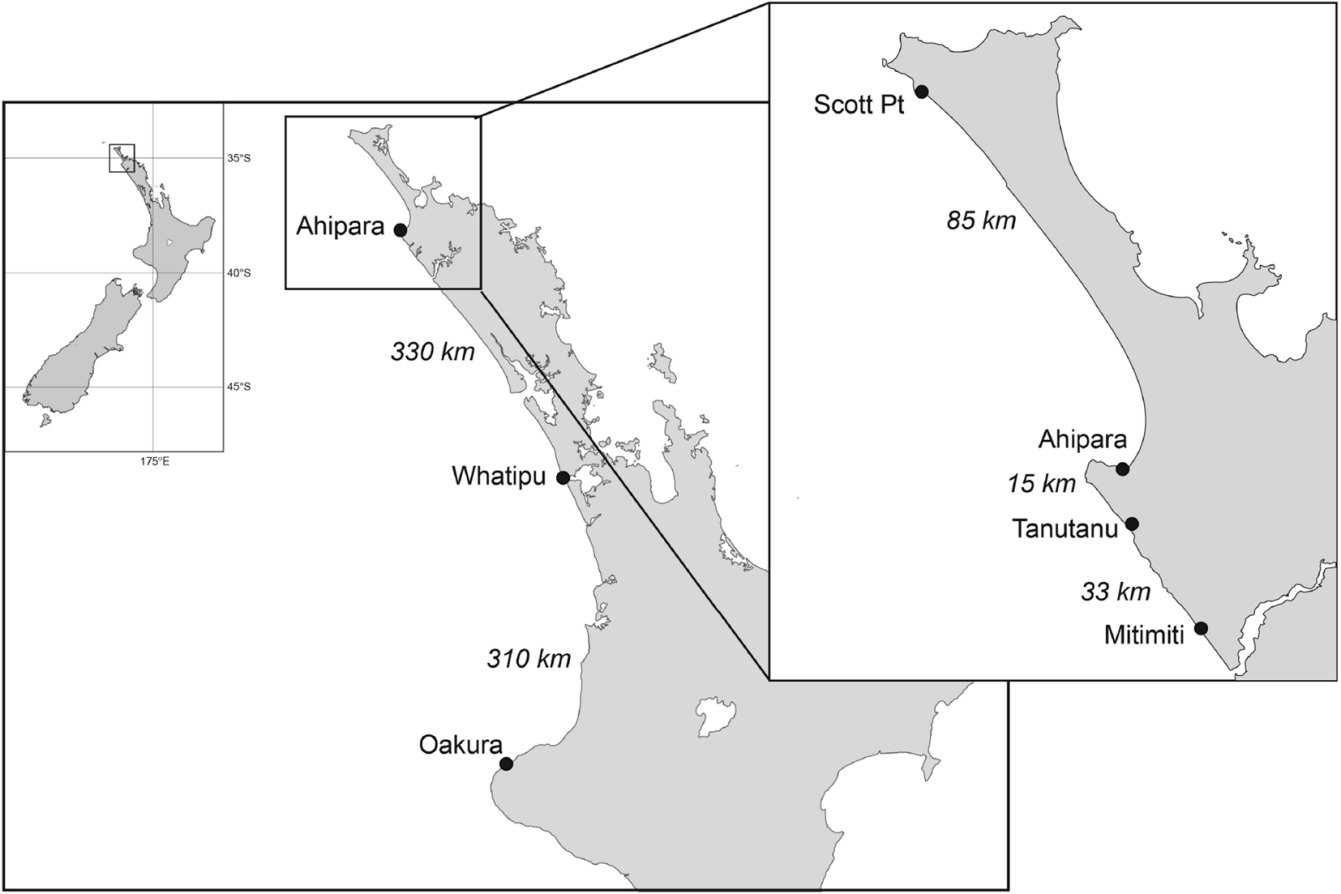
Location of sampling sites from which green-lipped mussels (*P. canaliculus*) were collected along the west coast of the North Island of New Zealand.

Juvenile (sexually immature) green-lipped mussels, *Perna canaliculus*, 14–20 mm shell length, were collected in January 2015 from six intertidal reef sites along the west coast of the North Island of New Zealand (Fig. 4, Table S1). At these sites mussels of this size are expected to have settled within the last twelve months^60^. Mussels were cleaned of epibiota and had a periostracum from umbo to shell edge. Mussels were stored in plastic zip lock bags and frozen at − 20 °C within 1 h of collection. In the laboratory, mussels were thawed and photographed to record shell length (Table S1) via ImageJ software. Using plastic tweezers, mussels were opened and the flesh removed and placed in tubes containing 4 mL of 95% EtOH for genetic analysis. Shells were stored in plastic vials and refrozen for ICP-MS elemental analysis. For these mussel samples we consider that the genetic data (see below) relate to the site from which the larvae originated (i.e., the source population) whereas the shell microchemistry data (see below) relate to the site at which the individual mussel has resided the longest (i.e., not necessarily the source population, but if the mussel in question is a self-recruit then the site at which the mussel resides is actually the source population).

### Microsatellite marker methods

#### DNA extraction and genotyping

Total DNA was extracted from mantle or gill tissue using Geneaid genomic DNA kits following manufacturer´s instructions. DNA concentrations and A260/A280 ratios were quantified using a NanoDrop ND-1000 (Thermo Scientific). Specimens were genotyped at 10 polymorphic microsatellite loci^61^*.* Microsatellite loci were PCR-amplified in 15 μL reactions containing 70 ng of DNA template, 0.5 units/μL *Taq* DNA polymerase, 67 mM Tris–HCl pH 8.8, 16 mM (NH_4_)SO_4_, 2 mM MgCl_2_, 0.2 mM dNTPs, 0.3 μM of Forward and Reverse primers and ddH_2_O to volume. PCR products were visualised with an automated sequencer (ABI PRISM 3730 DNA Sequencer, Applied Biosystems) with the GeneScan-500 (LIZ) internal size standard. The software GeneMarker V2.2.0 (SoftGenetics) was used to analyse electropherograms for allele scoring and alleles were binned with manual checking using the AutoBin program^62^. Genotyping artefacts (null alleles, stuttering) were assessed using the software Micro-Checker v.2.2.0.3^63^.

#### Analysis of genetic diversity

Number of alleles (*A*), observed (*H*_O_) and expected (*H*_E_) heterozygosities per site and the number of private alleles (*A*_P_) were calculated using GenePop v4.7 on the web (https://genepop.curtin.edu.au/). Analysis of allelic and genotypic differentiation, departure from Hardy–Weinberg equilibrium (HWE) and linkage disequilibrium (LD) were performed via GenePop using the Markov chain method and Fisher´s exact test^64^. False discovery rate (FDR) control^65^ was applied to *P*-values in all statistical analyses that included multiple comparisons. A principal coordinates analysis (PCoA) of allelic data with site as the grouping variable was carried in PRIMER using the MDS function^66^ to examine variation in the multilocus data set.

#### Assignment testing

To determine the most likely source of individuals at each site, self-assignment tests were performed in the software GeneClass2^67^ using a Monte-Carlo resampling approach and the leave-one-out procedure, in which the individual under consideration is removed during computations for their site of collection^68^. The probability of assignment was based on 10,000 simulated individuals and an exclusion threshold of *P* < 0.05. Individuals that were excluded from their site of collection were assigned to another sampled site when *P* > 0.1. For detection of first-generation migrant individuals, we used the ‘L_home’ likelihood computation method to allow for the possibility that not all source sites were sampled, which is highly likely in this situation. Individuals identified as first-generation migrants were removed from the data set, and the remaining sampled individuals were used as a reference group for further assignment tests to identify the most likely source site for each first generation migrant. Where the results indicated more than one possible sampled source site (*P* > 0.1), the individual was assigned to the site with the highest probability. Thus, self-assignments are ‘home’ individuals (self recruits), whereas the first-generation migrants are ‘away’ individuals (a migrant from one known site to another).

### Shell geochemistry

#### Preparation of shells

Individual shells were thawed and placed into 5 ml plastic vials and sonicated for 2 min before the water was drained. All plastic ware used to handle and store mussels after the sonication step was acid washed for 12 h in 10% HNO_3_ (Sigma-Aldrich trace element grade) and rinsed three times in de-ionised water. To standardise analyses amongst sites, only the left shell of each individual was analysed. Shells were cleaned^69^. To remove the periostracum and any remaining organic matter, each shell was soaked in a 1% H_2_O_2_ (Fisher Scientific trace analysis grade) solution buffered in 1 N NaOH (Sigma-Aldrich trace analysis grade) at 80 °C for 10 min. Shells were then rinsed by running under de-ionised water for 10s and stored in acid-washed plastic vials, until digestion.

#### Shell digestion and ICP-MS analyses

After weighing, shells were placed in Maxi-44 80 ml Teflon vessels and combined with 5 ml HNO_3_ (65%) and 1 ml HCl (37%). Shells were digested using a Milestone ETHOS-UP microwave digestion system. Digests were diluted with 50 ml dH_2_O and the final weights obtained. Final solutions were clear and the shells were completely dissolved. Solutions were diluted 10 × with matrix matched diluent and elemental ratios were then analysed using an Agilent 7700 ICP-MS (Santa Clara, CA, USA). Instrument settings were RF Power (W) = 1550, with a He gas flow rate of 4.3 L min^−1^. Based on the results of Dunphy et al. (2011)^21^ the elemental suite analysed consisted of: ^43^Ca, ^66^Zn, ^55^Mn, ^11^B, ^88^Sr, ^25^Mg, ^138^Ba, ^63^Cu, ^7^Li, ^60^Ni, ^47^Ti, and ^59^Co. Raw elemental concentrations were converted to molar concentrations i.e. mmol mol^−1^, and trace element:calcium (*TE*:Ca) ratios were calculated to standardise elemental concentrations.

#### Statistical analyses

To assess the efficacy of geochemical markers in discriminating amongst shells from the six sites, a quadratic discriminant function analysis (Q-DFA) was performed. A Q-DFA was selected due to differing covariance estimates amongst samples. Due to non-normality, data were rank transformed and the Q-DFA was performed on the rank values^70^. An imputation procedure was applied when the concentration of an element was below detection limits of the instrument. This consisted of substituting the recorded 0 value with half of the minimum value of that element recorded in all shells, from all sites combined (K. Ruggiero, Dept. Statistics, University of Auckland, pers. comm. 2015). The Q-DFA was performed stepwise by firstly running the analysis on all elemental ratios and recording the overall classification success. Then the elemental ratio with the least discriminatory influence was removed and the Q-DFA run again with the difference in classification success noted. This was repeated for all elemental ratios to identify the point at which no increase in the classification success was observed and thus which combination of elements would prove to be successful in discriminating amongst shells from the different sites. Mean canonical scores were calculated for shells from each site and a classification success table generated in order to gauge the success of the Q-DFA in assigning shells back to their site of collection based on elemental ratios. Areas of Receiver Operating Characteristic (ROC) curves were calculated to provide a measure of the sensitivity of the Q-DFA by comparing true positive classifications against the false positive classifications. Kruskall-Wallis ANOVAs were used to test elemental ratios of shells amongst the sites and the temporal samples. All analyses were carried out in JMP 13.0 software^71^.

### Site-specific clustering—genotypic and phenotypic variation in a spatial context

The software package Geneland v4.0.8 was employed to identify the number of clusters (*K*) within the six-site data set^34^. Geneland is a spatial Bayesian analysis method that can include not only genotypic data (co-dominant microsatellite variation) but also phenotypic data (shell geochemical variation) and geographic data (latitude and longitude). It is implemented in the R language code^72^. We conducted four separate sets of analyses in Geneland: (1) the genetic data only were analysed in a geospatial setting, (2) the shell geochemistry data only were analysed in a geospatial framework, (3) the three samples from Ahipara (January, February, March 2015) were analysed in a geospatial context as a separate test of temporal variation in shell geochemistry i.e., we tested the hypothesis that shell geochemistry varied enough amongst the three samples to permit their identification as *K* = 3 clusters in Geneland, and we also analysed all eight samples together in a geospatial context (the six spatial samples collected in January 2015 plus the February and March 2015 samples collected from Ahipara) to test the extent of temporal variability in the range of spatial variability, and (4) the genetic and the shell geochemistry data were analysed together in a geospatial framework. This stepwise approach permits identification of clustering within the genetics dataset, within the shell geochemistry dataset, and then within the combined genotypic and phenotypic dataset. Geneland assumes approximate Hardy–Weinberg equilibrium (HWE) and linkage equilibrium between loci for the genotypic data. Because molluscan populations are rarely in HWE and are often characterised by null alleles^39,73^ we accounted for this in the analysis using the ‘null alleles’ module. We employed the ‘spatial’ and ‘correlated allele frequencies’ modules because previous work has shown that allele frequencies in one population are similar to those in other neighbouring populations^39^. Finally, spatial uncertainty was set to zero because mussels are sessile and we know their exact site of collection. We ran multiple independent runs (100,000 iterations, thinning set at 100, minimum number of populations set to 1, maximum number of populations set to the maximum number of samples included in the analysis) to test for the number of clusters (*K*) and to ensure that the results converged. In all three cases (genotypic data, phenotypic data, genotypic plus phenotypic data) the results of these runs were assessed based on the frequency of the different *K* values and also on a ranking of the run-specific probability density values. For the genotypic data analysis we calculated the pairwise *F*_ST_ values between the different clusters to help quantify the extent of differentiation amongst clusters.

## Supplementary Information

Supplementary Information 1.

Supplementary Information 2.
