# Supplementary material for: Combining genotypic and phenotypic variation in a geospatial framework to identify sources of mussels in northern New Zealand
Source: Sci Rep. 2021 Apr 14;11:8196. doi: 10.1038/s41598-021-87326-4 (PMC8046997; doi:10.1038/s41598-021-87326-4)
Supplement: Supplementary file 1 — Supplementary Information 1. [file 41598_2021_87326_MOESM1_ESM.docx]

**Combining genotypic and phenotypic variation in a geospatial framework to identify sources of mussels in northern New Zealand**

SUPPLEMENTARY MATERIAL

Jonathan P. A. Gardner^1,*^, Catarina N.S. Silva^1,2^, Craig R. Norrie^3,4^, Brendon J. Dunphy^3^

^1^ – School of Biological Sciences, Victoria University of Wellington, Wellington 6140, New Zealand

^2^ – Present address - Centre for Sustainable Tropical Fisheries and Aquaculture, College of Science and Engineering, James Cook University, 1 James Cook Dr, Townsville, QLD 4811, Australia

^3^ – School of Biological Sciences, University of Auckland, Auckland 1142, New Zealand

^4^ – Present address - Hatfield Marine Science Center, Cooperative Institute for Marine Resources Studies, Oregon State University, Newport, OR 97365, USA

* author for correspondence – jonathan.gardner@vuw.ac.nz

Table S1. Sampling location, sample size and mean shell length of juvenile green-lipped mussels (*P. canaliculus*) collected in January 2015 from northern New Zealand. Sites are listed north to south. Name in parentheses indicates site code. Mussels were also collected from Ahipara (AHI) in February and March 2015 for analysis of shell geochemistry temporal variability using ICP-MS methods.

| Site | Grid coordinates | N analysed | Mean shell length  (± S.D) |
| --- | --- | --- | --- |
| Scott Point (SCO) | 34°31'34"S,  172°42'31"E | 50 | 16.3 (± 3.5) |
| Ahipara (AHI) | 35°10’40”S, 173°07’56”E | 50 | 16.4 (± 4.4) |
| AHI (February 2015) | 35°10’40”S, 173°07’56”E | 50 | 18.8 (±4.2) |
| AHI (March 2015) | 35°10’40”S, 173°07’56”E | 50 | 18.5 (±2.5) |
| Tanutanu Beach (TAN) | 35°12’14”S, 173°04’17”E | 50 | 20.1 (± 3.4) |
| Mitimiti Beach (MIT) | 35°25’51”S, 173°16’12”E | 50 | 19.0 (± 3.7) |
| Whatipu (WHA) | 37°02’41”S, 174°30’35”E | 50 | 14.5 (± 4.4) |
| Oakura Beach (OAK) | 39°06’33”S, 173°57’17”E | 50 | 17.0 (± 3.4) |

Table S2. Number of private alleles per site for each of 10 microsatellite loci.

|  | **Scott Point** | **Ahipara** | **Tanutanu** | **Mitimiti** | **Whatipu** | **Oakura** | **Total number of private alleles per locus** |
| --- | --- | --- | --- | --- | --- | --- | --- |
| Locus name  (number of alleles) |  |  |  |  |  |  |  |
| *Pcan1-25* (10) |  | 1 |  | 1 |  | 1 | 3 |
| *Pcan1-27* (16) | 1 |  | 1 | 1 |  | 1 | 4 |
| *Pcan1-29* (38) | 3 | 3 |  | 1 | 2 | 1 | 10 |
| *Pcan2-17*  (5) |  |  | 1 |  |  |  | 1 |
| *Pcan2-20* (13) |  |  |  | 1 | 1 |  | 2 |
| *Pcan2-60*  (5) |  | 1 |  | 1 |  |  | 2 |
| *Pcan6-17* (21) |  |  |  |  |  |  | 0 |
| *Pcan10-36* (9) |  |  |  |  |  | 1 | 1 |
| *Pcan10-44* (20) | 1 |  | 1 |  |  |  | 2 |
| *Pcan22-11* (23) |  | 2 |  | 2 |  |  | 4 |
| Total number of private alleles per site | 5 | 7 | 4 | 7 | 3 | 4 | Σ = 25 |

Table S3a. Results of the Geneclass2 analysis of the percentage of mussels collected from any given source site (rows) and assigned to all sites (columns). Correct assignments are marked on the diagonal and shown in bold italics.

Source Assigned site Total % assigned

site to non-source sites

________________________________________________________________________________

SCO AHI TAN MIT WHA OAK

Scott Point (SCO) ***29.8*** 2.1 4.3 2.1 51.1 10.6 70.2

Ahipara (AHI) 4.0 ***46.0*** 8.0 0.0 36.0 6.0 54.0

Tanutanu (TAN) 0.0 2.0 ***32.6*** 4.1 53.1 8.2 67.4

Mitimiti (MIT) 2.3 2.3 0.0 ***59.1*** 34.0 2.3 40.9

Whatipu (WHA) 10.5 2.6 2.6 0.0 ***79.0*** 5.3 21.0

Oakura (OAK) 6.1 4.1 2.0 0.0 34.7 ***53.1*** 46.9

Table S3b. Results of the Geneclass2 analysis of the number of first generation migrants (*F*_0_) from each source site to each destination site.

Source site Destination site Total

___________________________________________________________________________

SCO AHI TAN MIT WHA OAK

Scott Point (SCO) 2 1 1 4

Ahipara (AHI) 1 2 3

Tanutanu (TAN) 0

Mitimiti (MIT) 1 1 2

Whatipu (WHA) 1 1 2

Oakura (OAK) 1 1

Total 3 1 1 3 3 1 12

Table S4. Stepwise variable selection of elemental ratios within shells of juvenile green-lipped mussel, *Perna canaliculus*, collected from Scott Point, Ahipara (January, February, March 2015), Tanutanu Beach, Mitimiti, Whatipu and Oakura in 2015. Shaded rows denote the elemental ratios used in subsequent quadratic discriminant function analyses (Q-DFA).

| Elemental ratio  X:Ca | Stepwise cumulative % Classified | Additional classification provided by this elemental ratio |
| --- | --- | --- |
| Mg | 45.6 | - |
| B | 63.8 | 18.2 |
| Mn | 86.3 | 22.5 |
| Li | 89.9 | 3.6 |
| Ni | 94.2 | 4.3 |
| Co | 96.7 | 2.5 |
| Zn | 97.0 | 0.3 |
| Ba | 97.7 | 0.7 |
| Cu | 98.0 | 0.3 |
| Ti | 98.7 | 0.7 |
| Sr | 98.7 | 0 |

Table S5. Multiple testing results for shell geochemistry.

This is a very large excel spreadsheet and will be uploaded separately.

Table S6. Results of 20 independent Geneland runs to determine number of clusters (*K*) within the genotypic data set and to estimate pairwise *F*_ST_ values between/amongst clusters for each run.

| RUN | *K* |  | Cluster 1 | Cluster 2 |  |  |  | |
| --- | --- | --- | --- | --- | --- | --- | --- | --- |
| 1 | 2 | Cluster 1 | 0 |  |  |  |  | |
|  |  | Cluster 2 | 0.02667 | 0 |  |  |  | |
|  |  |  |  |  |  |  |  | |
| 2 | 3 |  | Cluster 1 | Cluster 2 | Cluster 3 |  |  | |
|  |  | Cluster 1 | 0 |  |  |  |  | |
|  |  | Cluster 2 | 0.00454 | 0 |  |  |  | |
|  |  | Cluster 3 | 0.02528 | 0.03554 | 0 |  |  | |
|  |  |  |  |  |  |  |  | |
| 3 | 2 |  | Cluster 1 | Cluster 2 |  |  |  | |
|  |  | Cluster 1 | 0 |  |  |  |  | |
|  |  | Cluster 2 | 0.0029 | 0 |  |  |  | |
|  |  |  |  |  |  |  |  | |
| 4 | 3 |  | Cluster 1 | Cluster 2 | Cluster 3 |  |  | |
|  |  | Cluster 1 | 0 |  |  |  |  | |
|  |  | Cluster 2 | 0.02725 | 0 |  |  |  | |
|  |  | Cluster 3 | 0.00713 | 0.02987 | 0 |  |  | |
|  |  |  |  |  |  |  |  | |
| 5 | 4 |  | Cluster 1 | Cluster 2 | Cluster 3 | Cluster 4 | |  |
|  |  | Cluster 1 | 0 |  |  |  | |  |
|  |  | Cluster 2 | 0.00532 | 0 |  |  | |  |
|  |  | Cluster 3 | 0.00809 | 0.00825 | 0 |  | |  |
|  |  | Cluster 4 | 0.02569 | 0.03554 | 0.02987 | 0 | |  |
|  |  |  |  |  |  |  | |  |
| 6 | 3 |  | Cluster 1 | Cluster 2 | Cluster 3 |  | |  |
|  |  | Cluster 1 | 0 |  |  |  | |  |
|  |  | Cluster 2 | 0.0014 | 0 |  |  | |  |
|  |  | Cluster 3 | 0.0001 | -0.0021 | 0 |  | |  |
|  |  |  |  |  |  |  | |  |
| 7 | 3 |  | Cluster 1 | Cluster 2 | Cluster 3 |  | |  |
|  |  | Cluster 1 | 0 |  |  |  | |  |
|  |  | Cluster 2 | 0.03554 | 0 |  |  | |  |
|  |  | Cluster 3 | 0.00454 | 0.02528 | 0 |  | |  |
|  |  |  |  |  |  |  | |  |
| 8 | 3 |  | Cluster 1 | Cluster 2 | Cluster 3 |  | |  |
|  |  | Cluster 1 | 0 |  |  |  | |  |
|  |  | Cluster 2 | 0.02528 | 0 |  |  | |  |
|  |  | Cluster 3 | 0.03554 | 0.00454 | 0 |  | |  |
|  |  |  |  |  |  |  | |  |
| 9 | 4 |  | Cluster 1 | Cluster 2 | Cluster 3 | Cluster 4 | |  |
|  |  | Cluster 1 | 0 |  |  |  | |  |
|  |  | Cluster 2 | 0.00809 | 0 |  |  | |  |
|  |  | Cluster 3 | 0.02987 | 0.02569 | 0 |  | |  |
|  |  | Cluster 4 | 0.00825 | 0.00532 | 0.03554 | 0 | |  |
|  |  |  |  |  |  |  | |  |
| 10 | 3 |  | Cluster 1 | Cluster 2 | Cluster 3 |  | |  |
|  |  | Cluster 1 | 0 |  |  |  | |  |
|  |  | Cluster 2 | 0.02528 | 0 |  |  | |  |
|  |  | Cluster 3 | 0.03554 | 0.00454 | 0 |  | |  |
|  |  |  |  |  |  |  | |  |
| 11 | 2 |  | Cluster 1 | Cluster 2 |  |  | |  |
|  |  | Cluster 1 | 0 |  |  |  | |  |
|  |  | Cluster 2 | 0.02667 | 0 |  |  | |  |
|  |  |  |  |  |  |  | |  |
| 12 | 3 |  | Cluster 1 | Cluster 2 | Cluster 3 |  | |  |
|  |  | Cluster 1 | 0 |  |  |  | |  |
|  |  | Cluster 2 | 0.00454 | 0 |  |  | |  |
|  |  | Cluster 3 | 0.02528 | 0.03554 | 0 |  | |  |
|  |  |  |  |  |  |  | |  |
| 13 | 2 |  | Cluster 1 | Cluster 2 |  |  | |  |
|  |  | Cluster 1 | 0 |  |  |  | |  |
|  |  | Cluster 2 | 0.0029 | 0 |  |  | |  |
|  |  |  |  |  |  |  | |  |
| 14 | 3 |  | Cluster 1 | Cluster 2 | Cluster 3 |  | |  |
|  |  | Cluster 1 | 0 |  |  |  | |  |
|  |  | Cluster 2 | 0.02725 | 0 |  |  | |  |
|  |  | Cluster 3 | 0.00713 | 0.02987 | 0 |  | |  |
|  |  |  |  |  |  |  | |  |
| 15 | 4 |  | Cluster 1 | Cluster 2 | Cluster 3 | Cluster 4 | |  |
|  |  | Cluster 1 | 0 |  |  |  | |  |
|  |  | Cluster 2 | 0.00532 | 0 |  |  | |  |
|  |  | Cluster 3 | 0.00809 | 0.00825 | 0 |  | |  |
|  |  | Cluster 4 | 0.02569 | 0.03554 | 0.02987 | 0 | |  |
|  |  |  |  |  |  |  | |  |
| 16 | 3 |  | Cluster 1 | Cluster 2 | Cluster 3 |  | |  |
|  |  | Cluster 1 | 0 |  |  |  | |  |
|  |  | Cluster 2 | 0.0014 | 0 |  |  | |  |
|  |  | Cluster 3 | 0.0001 | -0.0021 | 0 |  | |  |
|  |  |  |  |  |  |  | |  |
| 17 | 3 |  | Cluster 1 | Cluster 2 | Cluster 3 |  | |  |
|  |  | Cluster 1 | 0 |  |  |  | |  |
|  |  | Cluster 2 | 0.03554 | 0 |  |  | |  |
|  |  | Cluster 3 | 0.00454 | 0.02528 | 0 |  | |  |
|  |  |  |  |  |  |  | |  |
| 18 | 3 |  | Cluster 1 | Cluster 2 | Cluster 3 |  | |  |
|  |  | Cluster 1 | 0 |  |  |  | |  |
|  |  | Cluster 2 | 0.02528 | 0 |  |  | |  |
|  |  | Cluster 3 | 0.03554 | 0.00454 | 0 |  | |  |
|  |  |  |  |  |  |  | |  |
| 19 | 4 |  | Cluster 1 | Cluster 2 | Cluster 3 | Cluster 4 | |  |
|  |  | Cluster 1 | 0 |  |  |  | |  |
|  |  | Cluster 2 | 0.00809 | 0 |  |  | |  |
|  |  | Cluster 3 | 0.02987 | 0.02569 | 0 |  | |  |
|  |  | Cluster 4 | 0.00825 | 0.00532 | 0.03554 | 0 | |  |
|  |  |  |  |  |  |  | |  |
| 20 | 3 |  | Cluster 1 | Cluster 2 | Cluster 3 |  | |  |
|  |  | Cluster 1 | 0 |  |  |  | |  |
|  |  | Cluster 2 | 0.02528 | 0 |  |  | |  |
|  |  | Cluster 3 | 0.03554 | 0.00454 | 0 |  | |  |
|  |  |  |  |  |  |  | |  |
